# Brief Teaching Intervention Improves Medical Students’ Dermatology Diagnostic Skills and Comfort in Performing Dermatology Exams

**DOI:** 10.3390/healthcare12141453

**Published:** 2024-07-22

**Authors:** Cristina Ricco, Babar K. Rao, Amy S. Pappert, Kristen M. Coppola

**Affiliations:** 1 Department of Dermatology, Rutgers Robert Wood Johnson Medical School, Somerset, NJ 08873, USApapperas@rwjms.rutgers.edu (A.S.P.); 2 Department of Psychiatry, Rutgers Robert Wood Johnson Medical School, Piscataway, NJ 08854, USA

**Keywords:** dermatology education, medical student education, dermatology curriculum, dermatology teaching course, student-run clinic, skin of color

## Abstract

Background: Skin disease is a significant contributor to the global disease burden, with dermatologic health disparities adding to this burden. Internists, general practitioners, and other medical professionals often manage skin disease with limited exposure to dermatologic education in medical school. Objective: This study evaluated a brief educational intervention for medical students to improve dermatologic knowledge, diagnostic and communication skills, and comfort in performing dermatology-focused physical exams. A secondary focus of the intervention was to promote awareness of skin disease, detection, and prevention for patients with a variety of skin tones. Methods: Sixty-five first through fourth-year students at Rutgers RWJMS participated in a pre-test–post-test within-subject study. Students described images using open-ended responses followed by multiple-choice identification questions. Students watched a one-hour self-paced module created by a licensed dermatologist and completed a follow-up assessment. Results: At pre-test, descriptions were brief and often inaccurate but significantly improved post-intervention to include descriptors such as primary morphology and demarcation. Accuracy on diagnostic and management questions significantly improved and comfort in advising patients and performing dermatologic exams significantly increased. Conclusions: A low-cost, brief, self-paced module can augment dermatologic education for medical students while increasing exposure to multiple skin tone presentations of lesions.

## 1. Introduction

Dermatologic disease is one of the leading causes of global disease burden, costing the United States an estimated USD 75 billion in 2013 [1]. Skin cancer is also the most common cancer in the U.S., with 197,700 cases of melanoma (both in situ and invasive) estimated in 2022 [2]. Although dermatologists are most equipped to diagnose and manage skin conditions, more than 40% of the U.S. population lives in areas with limited dermatology access [3,4]. As a result, general practitioners must be able to handle cutaneous concerns and recognize potentially malignant features of skin disease [3,4]. Despite this need, many general practitioners and internists have expressed uncertainty regarding the management of dermatologic conditions [5] and have a low self-perceived ability for diagnostics, which was confirmed via poor performance on photographic evaluation [6]. Additionally, less than 40% of primary care residents felt like their medical school curriculum adequately prepared them to manage dermatologic conditions [7]. Although the incidence of skin cancers has continued to increase over the last decade [8,9], the mean total hours of dermatology instruction has only increased by one [10].

Few studies have characterized dermatologic education in U.S. medical schools. The mean number of hours of dermatology instruction over the four years of schooling is reported to be 12.6 (median,10; range 0–34) [10]. Cahn et al. surveyed 137 LCME institutions and found that only 16 of 135 (12%) had a specific dermatology course as a part of the preclinical curriculum, and 49 out of 135 (36%) schools incorporated dermatology as a part of a broader block [11]. A scoping review of dermatology teaching supported the efficacy of implementing didactic principles, such as the activation of previous knowledge and knowledge tests, in interventions [12]. Furthermore, the visual nature of dermatologic diseases necessitates imaged-based learning, which has produced significant improvements in OSCE performances and knowledge tests [13].

Dermatologist-led instruction on how to approach the evaluation of images can help students develop astute pattern recognition, especially for different skin tones. Only a small percentage of images used in medical schools depict examples of skin of color [14]. This finding is particularly important given that racial and ethnic minorities are more likely to be uninsured [15] and have a higher mortality rate from melanoma compared to white individuals, often due to a delay in detection or diagnosis [16]. Ensuring that students receive effective dermatology training in medical school can help fight both skin disease burden in the U.S. as well as dermatologic healthcare disparities.

Dermatologic education could provide benefits to all medical students, and it could especially be helpful to students who work in low-income communities, where patients may not be able to seek help from a specialist. The current research evaluates a brief, targeted, dermatologist-led educational intervention for use in preclinical curricula. Prior to this intervention, the Robert Wood Johnson Medical School (RWJMS) curriculum included a self-paced module that was graded for completion but did not assess students’ ability to identify and manage dermatologic conditions. The goal of the intervention was to improve the ability of RWJMS students who serve at the HIP-HOP-PC (Homeless and Indigent Population Health Outreach Project—Promise Clinic), a student-run, free primary care continuity clinic that serves the uninsured community of New Brunswick, New Jersey, to administer dermatology exams and advise on dermatologic conditions. A pre-test–post-test within-subject design was used to examine students’ skills in identifying common dermatologic conditions, general knowledge, and comfort in performing a dermatologic examination.

## 2. Methods

### 2.1. Participants

Sixty-five first-to-fourth-year medical students who attend the RWJMS and who work in the PC voluntarily participated in this study and completed both the pre-test and intervention. Fifty-seven percent completed the post-test. There were no significant differences in the final sample of students who completed the pre-test and post-test (n = 37) compared to those who began the study but did not complete the post-test (n = 28) regarding demographics (gender, grade level, and dermatology experience) and the total number of correct pre-test multiple-choice responses.

Participants were 56.8% female, with 24% White, 22% Asian, and 14% Black. Eighty-four percent were in the first or second year of medical school. Half of the sample had no prior relevant dermatologic experiences. For those reporting dermatologic experiences, 16% of students reported self-teaching of basic level dermatologic information, 10% reported personal experience as a patient, 10% reported clinical experience as a medical assistant or shadowing a dermatologist, and 8% had completed a dermatology elective at the RWJMS.

### 2.2. Materials

This study had a pre-test–post-test within-subject design (see Supplementary Materials). The educational intervention was a one-hour recorded lecture created by C.R. and A.S.P., which included clinical skills including how to take a skin history, properly describe and document skin lesions, and perform a physical dermatologic exam. In addition, detailed information on common diagnoses and their management was also included. Finally, healthcare maintenance information such as basic skincare and sun safety recommendations was discussed. The lecture was supported by PowerPoint Version 16.84.1 slides with images of lesions and other dermatologic diagnoses (e.g., verruca vulgaris, arthropod bites, and fungal infections). Students individually completed a pre-test, watched the podcast (65 verified views), and then completed the post-test via Qualtrics software, Version 2022 [17], at their convenience. Students received access to each part of the study once the previous part was completed.

### 2.3. Questionnaire

Image Identification Task: Five images were used to assess student knowledge of common dermatologic conditions, including a benign nevus, psoriasis plaque, verruca vulgaris, atopic dermatitis, and a papular rash. These images were chosen with approval from VisualDx [18]. Students first provided an open-ended description of the condition seen in each image. Using an open-ended format for response was important to assess baseline knowledge and gauge the level of detail that the student would have used to report the lesion to an attending physician without having the benefit of prompts. Following the open-ended description, students then responded to a multiple-choice question concerning the identification or description of the lesion/condition in the image (e.g., the lesion here is raised, scaly, and rough. The lesion is on the dorsal surface of the hand. This is a (A) verruca vulgaris; (B) actinic keratosis; (C) squamous cell carcinoma; (D) melanoma; (E) I do not know). Students’ knowledge of management of the condition was then assessed (e.g., what would you or your attending recommend for treatment of this lesion? (A) topical treatment; (B) systemic treatment; (C) labs (including a biopsy); (D) nothing; (E) I do not know). Finally, students’ confidence in their answer choice was assessed using a four-point Likert scale (1 = not at all confident; 4 = very confident). During the intervention’s teaching portion, different images were used so that students could not memorize the images and descriptors for use in the post-test. In addition, no information concerning the accuracy of open-ended responses or scores on multiple-choice items was given following the pre-test, enabling the use of identical images at pre- and post-test to assess learning. A final question with the same Likert scale was used to assess confidence in identifying lesions on different skin tones. Answers for all multiple-choice questions included “I do not know” to prevent score inflation due to guessing.

Sun protection and skincare: Students were asked to describe the Fitzpatrick scale in an open-ended question and then were given the definition and asked when advising patients on skincare, how important is it to educate them on sunscreen if their skin type is type I or II, III or IV, or V or VI, using a three-point scale (1 = low importance, 2 = important, 3 = very important). Students also used this scale to assess the importance of moisturizers for all skin types.

Comfort level with advising patients and performing exams: Students’ comfort with advising patients (defined as advising patients on their diagnosis, plan of care, and long-term therapies) and administering physical exams for dermatologic and other conditions that are typically covered in first- and second-year curricula (cardiovascular, gastrointestinal, neurological, and pulmonary) were assessed using a 5-point Likert scale (1 = very uncomfortable; 5 = very comfortable).

### 2.4. Coding and Transformations

The open-ended descriptions of the images were coded for accuracy in identifying primary morphology (1 point given if indicated papule, macule, plaque, patch, etc.), configuration (mention of shape, color, size, texture, or demarcation would each receive 1 point up to a maximum of 3), and location (1 point for distribution/arrangement) for a total of 5 possible “Description of Image” points. An open-ended description of the Fitzpatrick scale was assessed on a 4-point scale.

C.R. and K.M.C. analyzed the data using IBM Statistical Package for the Social Sciences, Version 28.0.1 [19]. Mean pre-test–post-test scores for description of image, confidence, and comfort were compared using paired *t*-tests. Pre-test–post-test accuracy in choosing diagnosis and treatment was compared using McNemar’s test for paired categorical responses. Repeated-measure analysis of variance (ANOVA) was used to compare the mean scores for advising patients on dermatologic matters and performing dermatologic exams compared to other systems (e.g., cardio). Paired *t*-tests were used for post hoc comparisons.

## 3. Results

Image identification task: The mean number of total correct descriptors per image and examples of descriptors are displayed in Table 1. There was a significant increase in the total points earned per description of an image from pre- to post-intervention for all images (*p* < 0.001).

**Figure 1:**
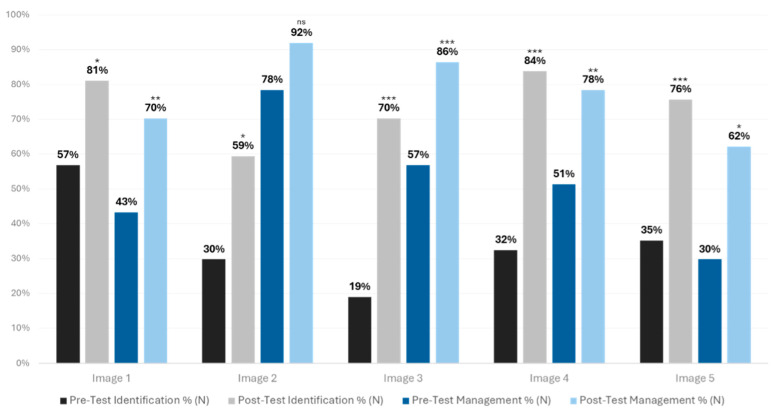
Percentage of students correctly answering diagnosis and management questions. Note: These paired dichotomous data (pre-test and post-test multiple-choice questions and pre-test and post-test treatment questions) were evaluated for significance using McNemar’s test (* *p* < 0.05, ** *p* < 0.01, *** *p* < 0.001; ns = non-significant).

For multiple-choice questions based on the identification of images and management decisions, the percentage of students with correct responses is displayed in Figure 1. There was a significant increase in number of identification questions answered correctly (Image 1: X^2^_36_ = 6.23, *p* = 0.01; Image 2: X^2^_36_ = 6.37, *p* = 0.01; Image 3: X^2^_36_ = 19, *p* = 0.00001; Image 4: X^2^_36_ = 17.19, *p* = 0.00003; Image 5: X^2^_36_ = 13.24, *p* = 0.0003). Significant increases in identifying the correct treatment of conditions were also found for four images (Q1: X^2^_36_ = 8.33, *p* = 0.004; Q3: X^2^_36_ = 11, *p* = 0.0009; Q4: X^2^_36_ = 7.14, *p* = 0.008; Q5: X^2^_36_ = 6.25, *p* = 0.012).

Mean confidence scores in multiple-choice answers were low at pre-test for all questions, and confidence increased after the intervention (i.e., Image 1: Mpre = 1.46 (1.24) Mpost = 2.7 (0.94); Image 2: Mpre = 1.3 (1) Mpost = 2.57 (0.99); Image 3: Mpre = 1.14 (1.06) Mpost = 2.49 (0.90); Image 4: Mpre = 1.24 (1.04) Mpost = 2.59 (0.87); Image 5: Mpre = 0.84 (0.87) Mpost = 2 (1.27)). All differences achieved statistical significance (Image 1: t_36_ = −6.94, *p* < 0.001; Image 2: t_36_ = −7.05, *p* < 0.001; Image 3: t_36_ = −7.76, *p* < 0.001; Image 4: t_36_ = −9.25, *p* < 0.001; Image 5: t_36_ = −6.06, *p* < 0.001).

Sun protection and skincare: At pre-test, students were unable to adequately describe the Fitzpatrick scale (M descriptors = 0.41 (0.76)). After the intervention, students received three of the four possible points (M = 3.16 (1.66); t_36_ = −8.80, *p* < 0.001). Student responses concerning the importance of recommending sunscreen varied by skin type. All students believed it was very important to recommend sunscreen skin types I–II at pre-test and post-test. Considering skin types III–IV, 67.6% (25/37) believed that it was very important to recommend sunscreen at pre-test, which increased to 89.2% (33/37) at post-test. For skin types V–VI, 59.5% (22/37) thought it important to advise sunscreen to use at pre-test and that number decreased to 45.9% (17/37) at post-test (consistent with the module content). Moisturizer use for skin barrier protection was rated as very important by most students (70.3%; 26/37), which increased to 94.6% (35/37) after the intervention.

**Figure 2:**
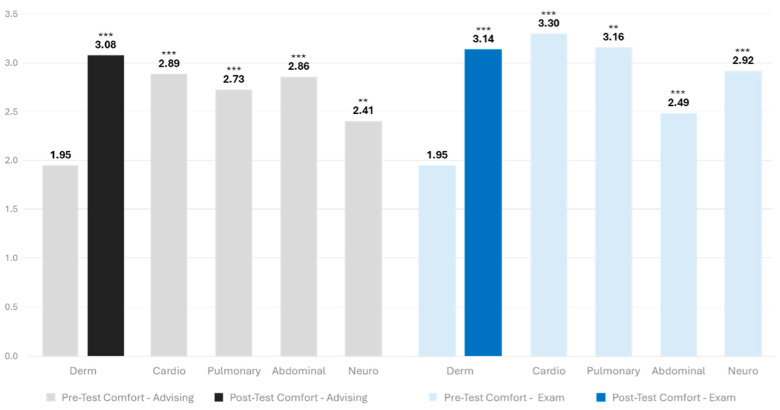
Comfort level with advising on dermatology and performing a dermatology exam compared to other specialties. Note: Mean scores were evaluated using repeated-measure ANOVA. Comfort levels ranged on a scale from 1 to 5. (** *p* < 0.01, *** *p* < 0.001).

Comfort level with advising patients and performing exams: Students’ mean comfort level for advising patients and performing dermatology exams was low at pre-test, while comfort levels at baseline for advising and performing other system-based exams was higher (means ranging from 2.41 to 3.33; see Figure 2).

At pre-test, there was an overall difference in students’ comfort in advising patients based on systems (F_36_ = 8.370; *p* < 0.001). Students had a significantly lower comfort level in advising patients on dermatologic conditions compared to advising on conditions of other systems. All post hoc paired comparisons for dermatology versus other systems were statistically significant (cardio: t_36_ = −5.33, *p* < 0.001; gastrointestinal t_36_ = −5.69, *p* < 0.001; pulmonary t_36_ = −4.62, *p* < 0.001; neurological t_36_ = −2.83, *p* = 0.008). At post-test, the overall ANOVA was statistically significant (F_36_ = 5.162; *p* = 0.002). The means for students’ comfort in advising on dermatology was higher than advising for other systems, and importantly, these differences did not achieve statistical significance (Figure 2).

There was also an overall difference in students’ comfort in performing different system-based physical exams at pre-test (F_36_ = 10.177; *p* < 0.001). Students had lower comfort levels in performing dermatologic examinations compared to those performing other system-based examinations. All post hoc paired comparisons for dermatology versus other systems were statistically significant (cardio: t_36_ = −6.45, *p* < 0.001; gastrointestinal t_36_ = −3.15, *p* = 0.003; pulmonary t_36_ = −5.81, *p* < 0.001; neurological t_36_ = −4.34, *p* < 0.001). At post-test, the overall ANOVA was statistically significant (F_36_ = 5.357; *p* = 0.002). The means for students’ comfort in performing dermatology examinations were higher than for performing gastrointestinal (abdominal) and neurological exams but lower than cardiovascular and pulmonary exams. The difference between dermatologic and abdominal examinations achieved statistical significance (t_36_ = −3.00, *p* = 0.005), while the other comparisons did not (post hoc comparisons were n.s.).

## 4. Discussion

Brief educational interventions can be effective for high-yield learning. Our study shows that didactic teaching in a one-hour lecture can efficiently provide a robust foundation of clinical knowledge. This is evidenced by improved performance on the post-test and increased confidence levels in ‘dermatologic language’, diagnosis, management, and examination. Students in our study also exhibited increased comfort in dermatology to the same degree as other systems in which they had already received lengthy training before the study. Students also showed increased knowledge concerning the importance of sun protection, moisturizer use, and skincare advisory.

Students’ performance on open-ended descriptions of clinical images significantly improved after the educational intervention. At pre-test, description questions were often left empty, whereas at post-test, descriptions were robust, such as “dark brown, 6–7 cm fully demarcated patch across the bilateral popliteal regions”, which acknowledges primary morphology, configuration, and location/distribution. By understanding the principles of dermatologic terminology, students were able to improve their diagnostic capabilities. The intervention also helped students differentiate between conditions that may appear similar to the untrained eye. For the fourth multiple-choice question provided during the pre-test, 32.43% of students selected psoriasis, and 32.43% selected eczema, whereas at post-test, 83.78% selected the correct answer (eczema).

At post-test, students improved their knowledge of the importance of sunscreen use for darker skin tones to prevent the development of pigment disorders, photoaging, and basal cell carcinoma [20,21]. Current evidence is unclear as to whether sunscreen use decreases cancer-related mortality in darker skin types [20], but this does not mean that no benefit exists, and further investigation on this topic is warranted. In addition, at post-test, students recognized the importance for all skin types to moisturize to protect the skin barrier and reduce the frequency of diseases like atopic dermatitis [22]. Students also gained exposure to diverse presentations as the materials in our study included the representation of various skin tones.

The measures used in this study allowed us to assess students’ abilities to apply their knowledge in a clinical setting. The use of various clinical images to train students in pattern recognition was a simple way to increase knowledge levels and performance. Our students were able to correctly use dermatologic terminology to characterize cutaneous observations, which will in turn improve diagnostic capabilities and assist students and residents when providing inpatient consultation. They were also able to identify the next steps in management for common conditions that they will likely see during their careers as students and physicians.

Secondly, we also observed that because the students developed a strong knowledge base, their comfort with advising patients on dermatologic conditions and performing dermatologic examinations improved post-intervention to the point that they were as comfortable with the dermatology exam as they were with cardiovascular, pulmonary, abdominal, and neurological exams. Because of this intervention, the PC has now adopted a dermatology clinic in which students can practice dermatologic care.

Limitations to this study include the shortened time frame of assessment for the retention of information, and longitudinal follow-up could strengthen the reliability of our findings. In addition, external validity could be improved by expanding the distribution of students as this intervention was targeted to first- and second-year students as well as students who volunteered in an outpatient clinic setting. Future studies should also consider an in-person component to assess examination skills.

Though one hour of lecture time cannot address all the fundamentals of dermatology, this method of teaching was successful and low-cost, and it may be adaptable for a larger lecture series. Competition for curriculum time can be a barrier to incorporating dermatology instruction [10]; however, using a self-paced module, educators can provide a foundation that can be built upon during clinical rotations. Using diverse imagery and focusing on the presentation of dermatologic conditions for patients in local markets, medical schools can equip students to participate in well-rounded patient care.

## Figures and Tables

**Table 1:** Mean number (SD) of correct descriptors per image (morphology, configuration, and location/arrangement).

Image	Pre-Test Descriptor	Post-Test Descriptor	T_(36)_	*p* Value	Pre-Test Descriptor Examples	Post-Test Descriptor Examples
1	1.11 (SD 1.28)	3 (SD 1.18)	−8.4	<0.001	- brown - circular	- 2 pts - macule - dark brown
2	1.05 (SD 1.41)	3.03 (SD 1.50)	−7.17	<0.001	- asymmetric - scaly	- 5 pts - plaque - bright pink - poorly demarcated - scaly - a single lesion
3	0.7 (SD 1.10)	2.65 (SD 1.40)	−7.75	<0.001	- raised - single lesion	- 4 pts - papule - scaly - verrucous - a single/one
4	0.76 (SD 1.34)	2.11 (SD 1.59)	−5.02	<0.001	- flat - scaly - erythematous	- 3 pts - patches - brown - flexor surfaces of bilateral knees
5	0.84 (SD 1.26)	2.76 (SD 1.30)	−9.64	<0.001	- pink - scattered lesions - raised	- 5 pts - papules - pink - round - pea-sized - multiple scattered lesions - over the back

## Data Availability

Data are contained within the article and Supplementary Materials.

## Supplementary Materials

The following supporting information can be downloaded at: https://www.mdpi.com/article/10.3390/healthcare12141453/s1, Dermatological Education Post-Test and Dermatological Education Pre-Test.
