# Supplementary material for: Brief Teaching Intervention Improves Medical Students’ Dermatology Diagnostic Skills and Comfort in Performing Dermatology Exams
Source: Healthcare (Basel). 2024 Jul 22;12(14):1453. doi: 10.3390/healthcare12141453 (PMC11276251; doi:10.3390/healthcare12141453)
Supplement: Supplementary file 1 [file healthcare-12-01453-s001.zip › HC - pretest _compressed.pdf]

Dermatological Education Pre-Test

Start of Block: 1/3 parts

Q31 Thank you for participating in our survey. This survey should take less than 10 minutes to complete. There are 3 parts to our survey with 32 total questions, not including the code identifier or demographic questions (optional). Results from the survey will be reported only in aggregate form.   This survey aims to assess Promise Clinic student doctors' comfort level and knowledge of dermatology. If you have questions about this study, please contact Dr. Babar Rao at raobk@rwjms.rutgers.edu.   There is a downloadable pdf of the consent form attached. Please review the consent form, and then select whether you agree or disagree to participating in the survey below.

Consent Form

Q67 I have read and understood the consent form and information above. I understand my participation is voluntary and am free to withdraw at any time. I voluntarily agree to participate in this study.

○ I agree (1)

○ I disagree (4)

Skip To: End of Survey If I have read and understood the consent form and information above. I understand my participation... = I disagree

Skip To: Q1 If I have read and understood the consent form and information above. I understand my participation... = I agree

---

Page Break

Q1 Please use this as your code for the pre and post test: (write it down!)
What is your favorite color? What is the name of your favorite animal? What is your favorite number?
Example: pinkelephant11

____

End of Block: 1/3 parts

Start of Block: Block 2

Q45 For the following set of questions, you will see 5 photos and be asked to use dermatologic descriptors to describe each one. If you do not know the answer, you can leave it blank and advance to the next questions based on the same photo.

Q19 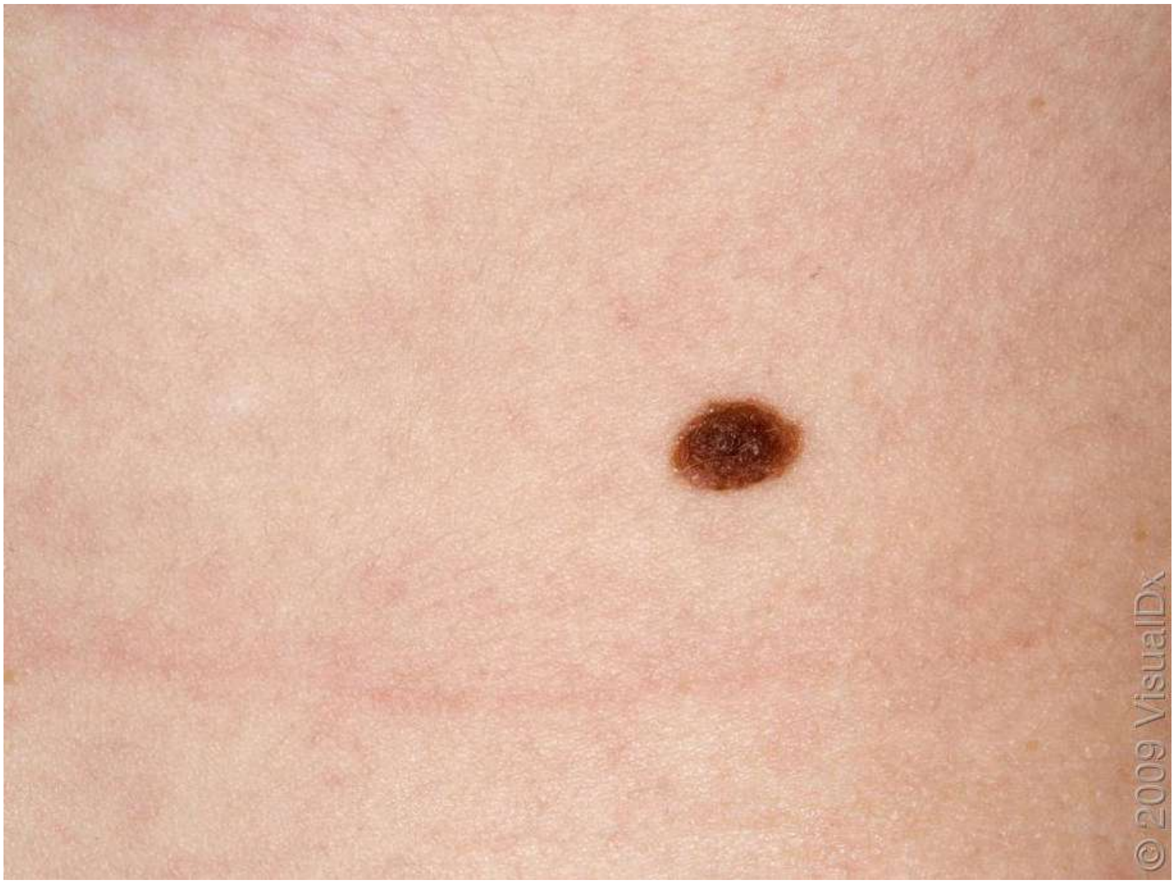

Q66 What words would you use to describe this lesion? If you do not know, you can leave this blank.

____

____

____

____

____

Page Break

Q69

Q20
Which descriptor best matches this nevus?

- ○ Dysplastic (1)
- ○ Benign (2)
- ○ Malignant (3)
- ○ I do not know (4)

Q75 What would you or your attending recommend for treatment of this lesion?

- ○ Topical Treatment (1)
- ○ Systemic Treatment (2)
- ○ Labs (including a biopsy) (3)
- ○ Nothing (4)
- ○ I do not know (5)

Skip To: Q43 If What would you or your attending recommend for treatment of this lesion? = I do not know

---

Page Break

Q42 How confident are you in your answer choices?

	Not at all confident (1)	Slightly confident (2)	Moderately confident (4)	Very confident (5)
I am... (1)	○	○	○	○

Page Break

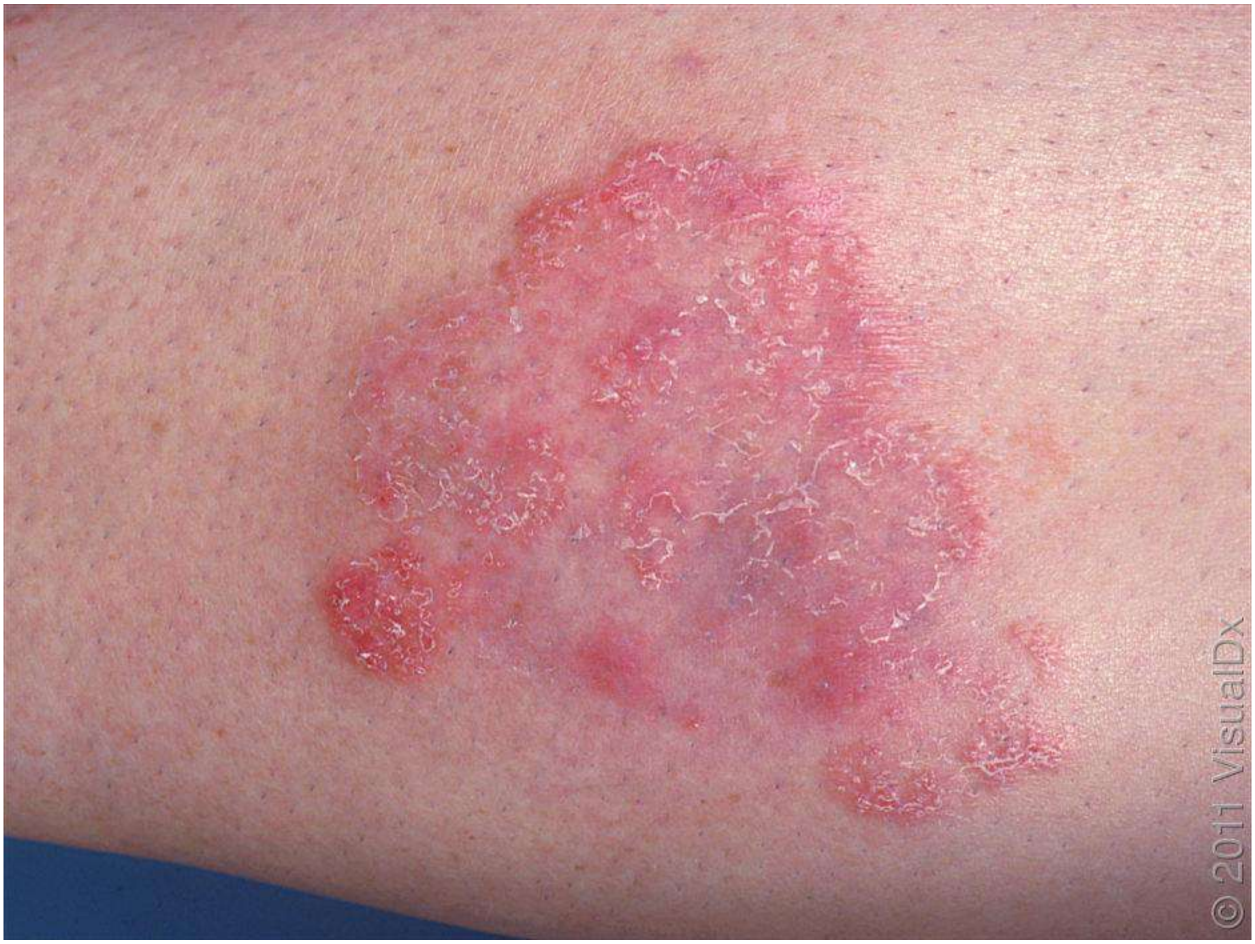

Q43

Q22 What words would you use to describe this lesion? If you do not know, you can leave this blank.

____

____

____

____

____

Page Break

Q62

Q44 The lesion is raised, crusted, and erythematous. This is an example of a:

- ◯ Macule (1)
- ◯ Patch (2)
- ◯ Plaque (3)
- ◯ Papule (4)
- ◯ I do not know (5)

Q76 What would you or your attending recommend for treatment of this lesion?

- ◯ Topical Treatment (1)
- ◯ Systemic Treatment (2)
- ◯ Labs (including a biopsy) (3)
- ◯ Nothing (4)
- ◯ I do not know (5)

Skip To: Q23 If What would you or your attending recommend for treatment of this lesion? = I do not know

---

Q51 How confident are you in your answer choices?

	Not at all confident (1)	Slightly confident (2)	Moderately confident (3)	Very Confident (4)
I am... (1)	○	○	○	○

Page Break

Q23

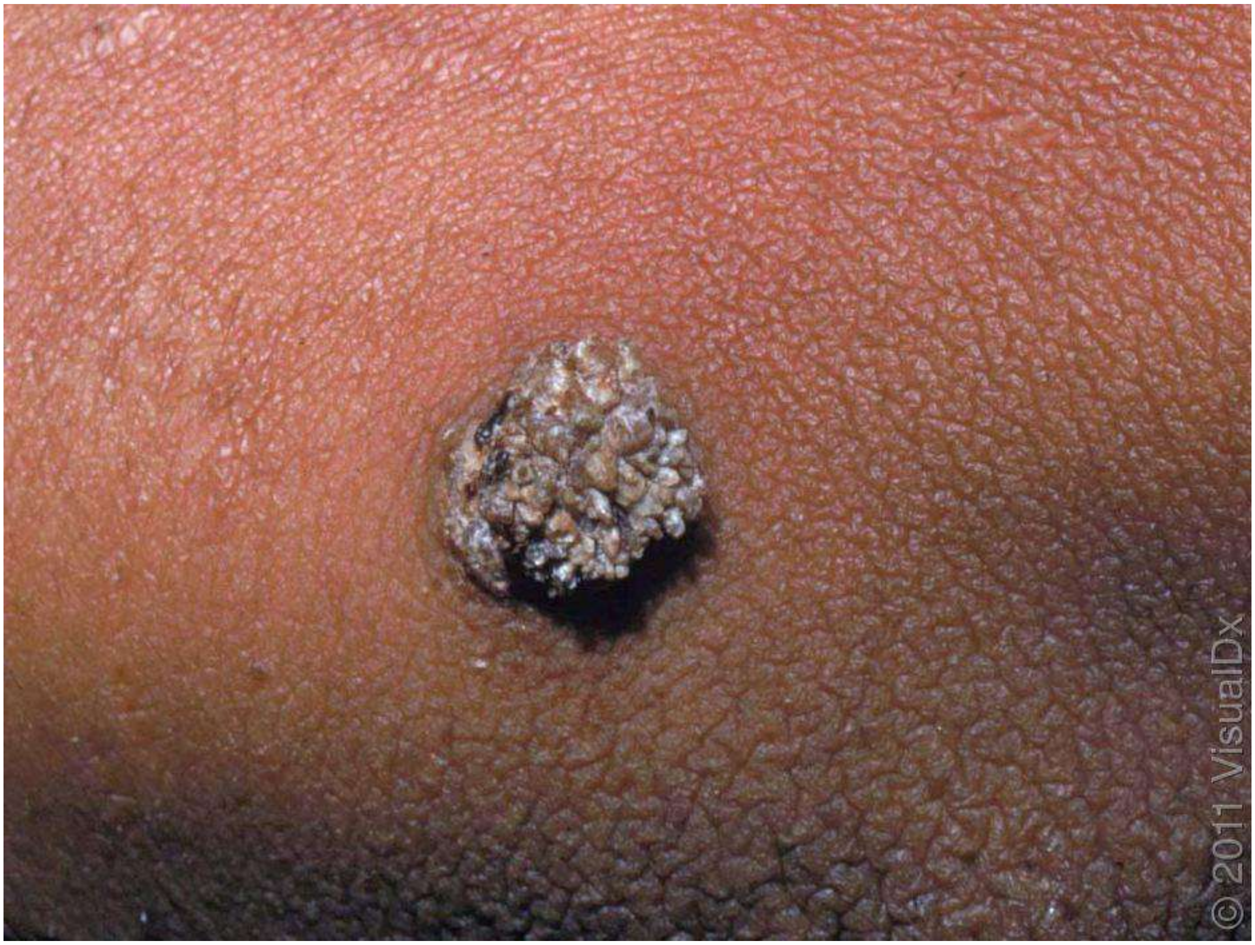

Q54 What words would you use to describe this lesion? If you do not know, you can leave this blank.

____

____

____

____

____

Page Break

Q53

Q24 The lesion here is raised, scaly. and rough. The lesion is on the dorsal surface of the hand. This is a:

- ○ Verruca Vulgaris (1)
- ○ Actinic Keratosis (2)
- ○ Squamous Cell Carcinoma (3)
- ○ Melanoma (4)
- ○ I do not know (5)

Q78 What would you or your attending recommend for treatment of this lesion?

- ○ Topical Treatment (1)
- ○ Systemic Treatment (2)
- ○ Labs (including a biopsy) (3)
- ○ Nothing (4)
- ○ I do not know (5)

Skip To: Q25 If What would you or your attending recommend for treatment of this lesion? = I do not know

Page Break

Q52 How confident are you in your answer choices?

	Not at all confident (1)	Slightly confident (2)	Moderately confident (3)	Very confident (4)
I am... (1)	○	○	○	○

Page Break

Q25

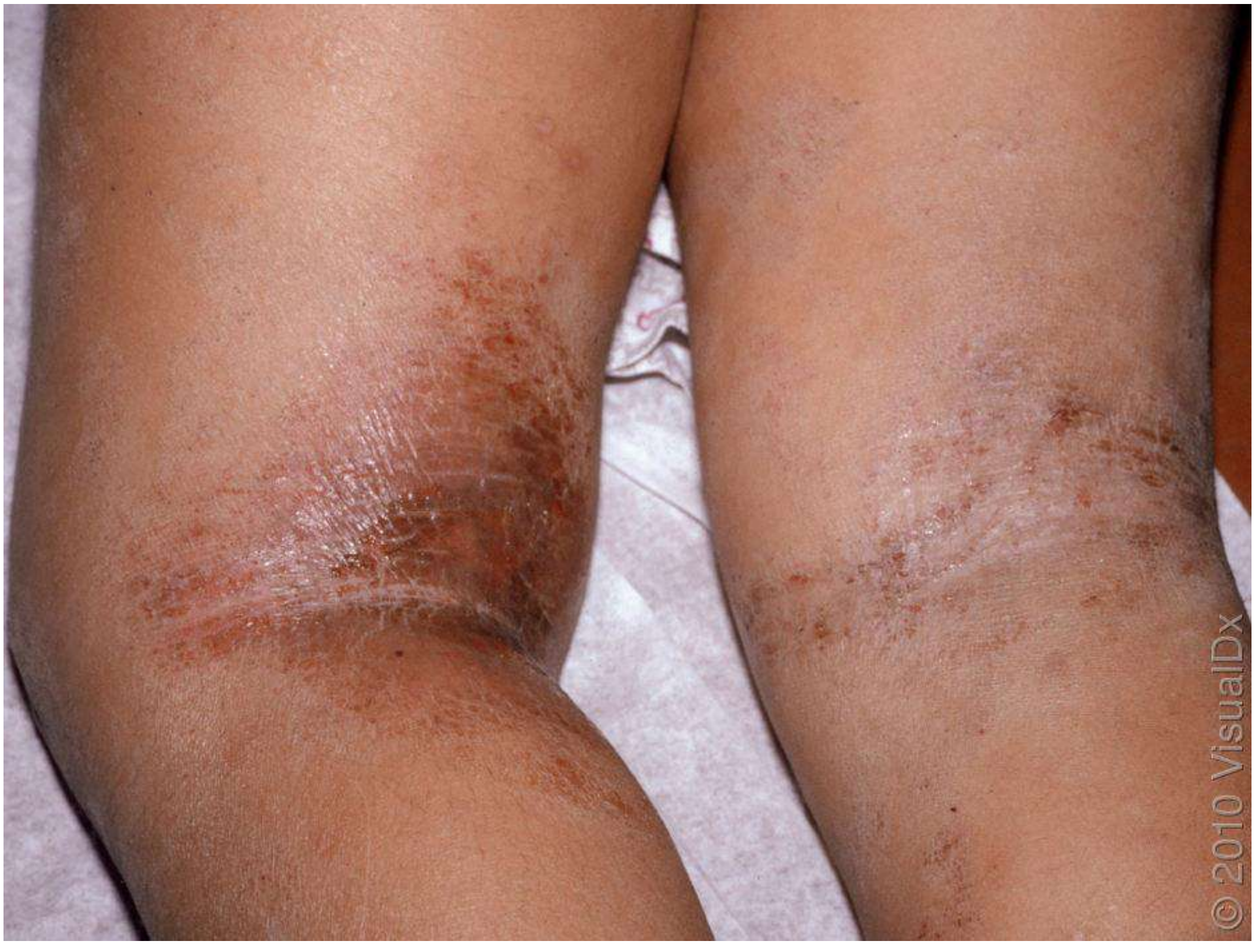

Q55 What words would you use to describe this lesion? If you do not know, you can leave this blank.

Page Break

Q57

Q26 The lesions are flat, crusted, itchy, and erythematous. They are present on the patient's popliteal fossa and antecubital fossa. This is an example of:

- ◯ Psoriasis (1)
- ◯ Eczema (2)
- ◯ Ringworm (3)
- ◯ Rosacea (4)
- ◯ I dont know (5)

Q79 What would you or your attending recommend for treatment of this lesion?

- ◯ Topical Treatment (1)
- ◯ Systemic Treatment (2)
- ◯ Labs (including a biopsy) (3)
- ◯ Nothing (4)
- ◯ I do not know (5)

Skip To: Q27 If What would you or your attending recommend for treatment of this lesion? = I do not know

Q56 How confident are you in your answer choices?

	Not at all confident (1)	Slightly confident (2)	Moderately confident (3)	Very confident (4)
I am... (1)	○	○	○	○

Page Break

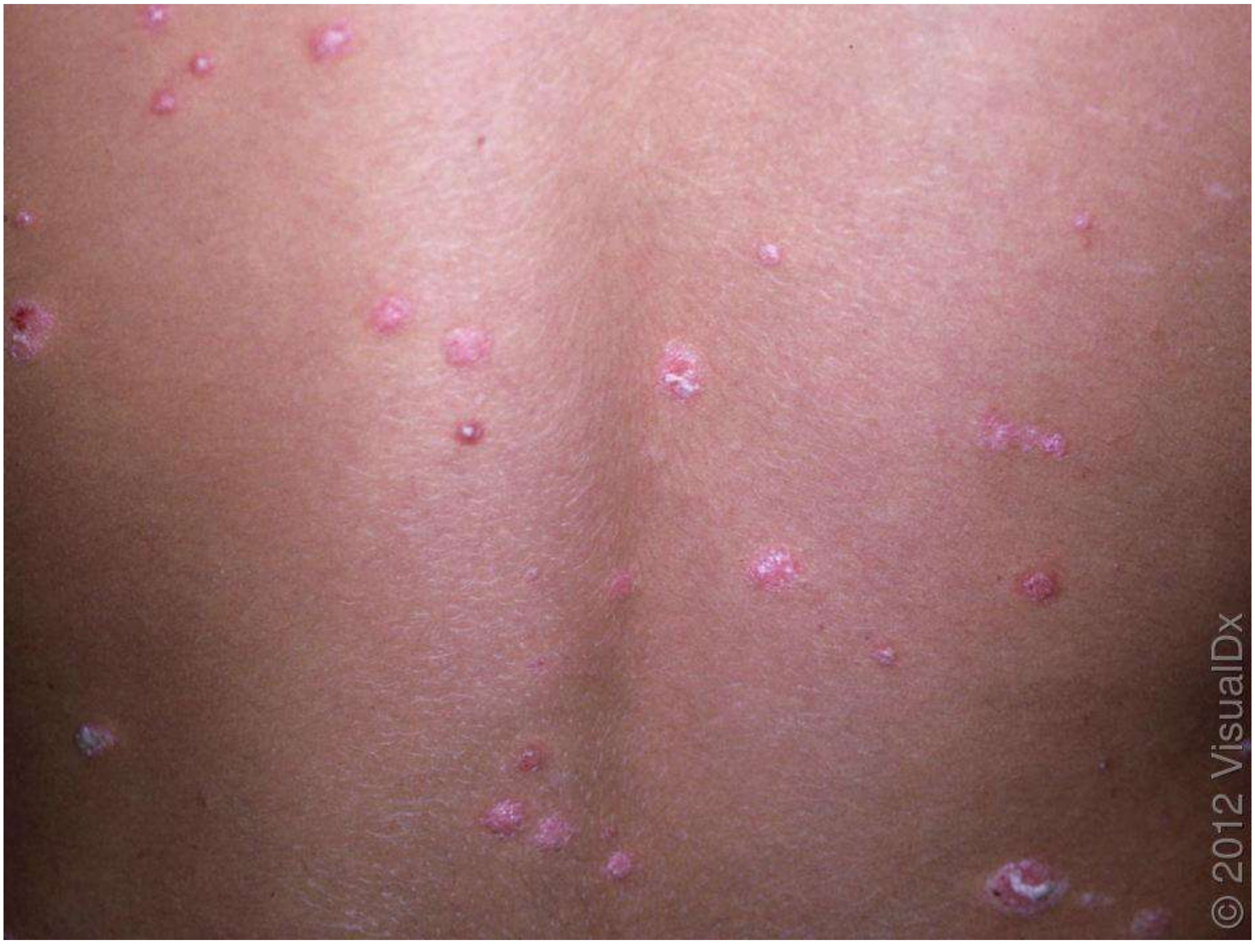

Q27

Q59 What words would you use to describe this lesion? If you do not know, you can leave this blank.

Page Break

Q28 I would describe each individual lesion as a:

- Erythematous macule (1)
- Pigmented nodule (2)
- Vesicle (3)
- Erythematous papule (4)
- I do not know (5)

Q80 What would you or your attending recommend for treatment of this lesion?

- Topical Treatment (1)
- Systemic Treatment (2)
- Labs (including a biopsy) (3)
- Nothing (4)
- I do not know (5)

Skip To: Q70 If What would you or your attending recommend for treatment of this lesion? = I do not know

Q60 How confident are you in your answer choices?

	Not at all confident (1)	Slightly confident (2)	Moderately confident (3)	Very confident (6)
I am... (1)	○	○	○	○

Q70 How confident are you in identifying lesions on different skin tones?

	Not at all confident (1)	Slightly confident (2)	Moderately confident (3)	Very confident (6)
I am... (1)	○	○	○	○

Page Break

Q63 In this section (2/3), you will have 6 questions based on skincare and sun protection.

What is the Fitzpatrick Scale?

____

____

____

____

____

Page Break

Q29 The Fitzpatrick Scale is a classification system for the way one's skin reacts to UV light. It ranges from Type 1, skin that burns easily (fairest complexion), to Type 6, skin that rarely burns (darkest complexion).

When advising patients on skincare, how important is it to educate them on sunscreen if their skin type is:

	Low Importance (2)	Important (4)	Very Important (5)
1-2 (1)	○	○	○
3-4 (2)	○	○	○
5-6 (3)	○	○	○

Q65 Have you ever seen a dermatologist?

○ Yes (1)

○ No (2)

○ I do not see a dermatologist (4)

○ Prefer not to answer (5)

Q64 Has your dermatologist advised you on using sunscreen?

- Yes (1)
- No (2)
- I do not see a dermatologist (3)
- I do not remember (6)
- Prefer not to answer (4)

Q73 Has your dermatologist advised you on using moisturizer?

- Yes (1)
- No (2)
- I do not see a dermatologist (3)
- I do not remember (5)
- Prefer not to answer (4)

Q81 How important do you think moisturizers are for all skin types?

	Low importance (2)	Important (4)	Very Important (5)
It is: (2)	○	○	○

End of Block: Block 2

Start of Block: 2/3 part

Q71 This is the last section of the survey that will consist of 5 opinion-based questions.

Page Break

Q3 As a student in the Promise Clinic, you advise patients on their diagnosis, plan of care, and long term therapies. Please evaluate whether you agree or disagree with the following statements using the Likert Scale provided.

How comfortable are you advising patients on the following conditions:

	Very uncomfortable (1)	Uncomfortable (2)	Neutral (3)	Comfortable (4)	Very Comfortable (5)
cardiovascular (1)	o	o	o	o	o
dermatologic (15)	o	o	o	o	o
gastrointestinal (3)	o	o	o	o	o
neurological (5)	o	o	o	o	o
pulmonary (2)	o	o	o	o	o

Q82 How comfortable are you administering the following exams?

	Very uncomfortable (1)	Uncomfortable (2)	Neutral (3)	Comfortable (4)	Very Comfortable (5)
cardiovascular (1)	○	○	○	○	○
dermatologic (15)	○	○	○	○	○
abdominal (3)	○	○	○	○	○
neurological (5)	○	○	○	○	○
pulmonary (2)	○	○	○	○	○

Q37 I have received an adequate amount of instruction and education through RWJMS or Promise Clinic on the following systems:

	Strongly Disagree (1)	Disagree (2)	Neither agree nor disagree (3)	Agree (4)	Strongly Agree (5)
cardiovascular (2)	○	○	○	○	○
dermatologic (9)	○	○	○	○	○
gastrointestinal (5)	○	○	○	○	○
neurological (7)	○	○	○	○	○
pulmonary (4)	○	○	○	○	○

Q83 How much time do you think should be given to dermatologic education?

	Lecture Hours<br>0 1 3 4 5 7 8 9 11 12 13 15 16 17 19 20
RWJMS ()	
Promise Clinic ()	

Q41 How important is it for you to provide a dermatology assessment for your patients in Promise Clinic?

	Not Important at all (1)	Low Importance (2)	Neutral (3)	Important (4)	Very Important (5)
It is... (1)	○	○	○	○	○

End of Block: 2/3 part

Start of Block: Block 3

Q2
I am a:(if you are a PhD or gap year student please select your highest level of medical school instruction)

○ M1 (1)

○ M2 (2)

○ M3 (3)

○ M4 (4)

Q49 Please briefly list any experiences you have with clinical dermatology or knowledge you may have learned outside of school (or inside of school):

____

Q85 The following questions are optional. You can choose "Prefer not to Answer" if you wish to skip them.

Q34 What is your gender identity?

- ○ Female (1)
- ○ Male (2)
- ○ Nonbinary (4)
- ○ Other (5) ____
- ○ Prefer not to answer (6)

Q46 What race do you identify with: (you may select more than one)

- ▢ Black/African American (1)
- ▢ White/Caucasian (2)
- ▢ Asian (3)
- ▢ American Indian or Alaska Native (4)
- ▢ Native Hawaiian or other Pacific Islander (5)
- ▢ Other (6) ____
- ▢ Prefer not to answer (7)

Q47 Are you of Hispanic/Latino origin?

- ○ Yes (1)
- ○ No (2)
- ○ Prefer not to answer (4)

End of Block: Block 3
